# Phenotypic Variation in Two Siblings Affected with Shwachman-Diamond Syndrome: The Use of Expert Variant Interpreter (eVai) Suggests Clinical Relevance of a Variant in the KMT2A Gene

**DOI:** 10.3390/genes13081314

**Published:** 2022-07-23

**Authors:** Ibrahim Taha, Federica De Paoli, Selena Foroni, Susanna Zucca, Ivan Limongelli, Marco Cipolli, Cesare Danesino, Ugo Ramenghi, Antonella Minelli

**Affiliations:** 1 Department of Molecular Medicine, University of Pavia, 27100 Pavia, Italy; ibrahimai.taha01@universitadipavia.it (I.T.); selena.foroni01@universitadipavia.it (S.F.); cesare.danesino@unipv.it (C.D.); 2 enGenome S.R.L., 27100 Pavia, Italy; fdepaoli@engenome.com (F.D.P.); szucca@engenome.com (S.Z.); ilimongelli@engenome.com (I.L.); 3 Centro Fibrosi Cistica, Azienda Ospedaliera Universitaria Integrata Verona, 37100 Verona, Italy; marco.cipolli@aovr.veneto.it; 4 Department of Pediatric and Public Health Sciences, University of Torino, 10126 Torino, Italy; ugo.ramenghi@unito.it

**Keywords:** Shwachman-Diamond Syndrome, *SBDS*, *KMT2A*, whole-exome sequencing, dual molecular diagnosis, eVai

## Abstract

Introduction. Shwachman-Diamond Syndrome (SDS) is an autosomal-recessive disorder characterized by neutropenia, pancreatic exocrine insufficiency, skeletal dysplasia, and an increased risk for leukemic transformation. Biallelic mutations in the *SBDS* gene have been found in about 90% of patients. The clinical spectrum of SDS in patients is wide, and variability has been noticed between different patients, siblings, and even within the same patient over time. Herein, we present two SDS siblings (UPN42 and UPN43) carrying the same *SBDS* mutations and showing relevant differences in their phenotypic presentation. Study aim. We attempted to understand whether other germline variants, in addition to *SBDS*, could explain some of the clinical variability noticed between the siblings. Methods. Whole-exome sequencing (WES) was performed. Human Phenotype Ontology (HPO) terms were defined for each patient, and the WES data were analyzed using the eVai and DIVAs platforms. Results. In UPN43, we found and confirmed, using Sanger sequencing, a novel de novo variant (c.10663G > A, p.Gly3555Ser) in the *KMT2A* gene that is associated with autosomal-dominant Wiedemann–Steiner Syndrome. The variant is classified as pathogenic according to different in silico prediction tools. Interestingly, it was found to be related to some of the HPO terms that describe UPN43. Conclusions. We postulate that the *KMT2A* variant found in UPN43 has a concomitant and co-occurring clinical effect, in addition to *SBDS* mutation. This dual molecular effect, supported by in silico prediction, could help to understand some of the clinical variations found among the siblings. In the future, these new data are likely to be useful for personalized medicine and therapy for selected cases.

## 1. Introduction

Shwachman-Diamond Syndrome (SDS) is an autosomal-recessive (AR) multi-systemic rare disease characterized by neutropenia, pancreatic exocrine insufficiency associated with steatorrhea and growth failure, skeletal dysplasia with short stature, and an increased risk of bone marrow aplasia or leukemic transformation. These are hallmark features that must be fulfilled to meet the SDS diagnosis. Other aspects, including hepatic complications, cardiac involvements, endocrine dysfunctions, behavioral and cognitive function abnormalities, and ocular, dental, and dermal manifestations are also observed in patients [1,2,3,4,5]. 

Biallelic pathogenic variants in the *SBDS* gene are found in >90% of SDS patients [6]. Recently, pathogenic variants in *DNAJC21*(AR), *EFL1* (AR), and *SRP54* (AD) have been reported in SDS or SDS-like phenotypes in a few cases [7,8,9,10,11,12]. However, despite the fact that *SBDS* and *EFL1* mutations have been associated with SDS1 (OMIM #260400) and SDS2 (OMIM#617941), respectively, only *SRP54* has been associated with SDS-like conditions (OMIM#618752). Conversely, *DNAJC21* has been associated with bone marrow failure syndrome 3 (OMIM#617052).

The clinical spectrum of patients affected with SDS is wide [4]. Phenotypic variability has been noticed between patients, siblings, and even within the same patient over time, making clinical diagnosis challenging in some cases [13]. For instance, hematological abnormalities are extremely common and may range from neutropenia, found in 88–100% of patients, to hematological malignancies including myelodysplastic syndrome (MDS) or acute myeloid leukemia (AML), which have been reported in about 17–35% of cases [13,14,15,16]. Pancreatic insufficiency is very common and found in 95% of patients. Additionally, failure to thrive, related to pancreatic insufficiency and feeding difficulties, has also been observed frequently (86.1%) [17]. On the other hand, skeletal defects, found in about 80.5% of SDS patients, are varied and may progress over time; they can also be widespread or localized, with variable degrees of severity [17].

Cardiac involvement, often reported as mild, is present in 43.5% of patients. Neuro-psychosocial issues related to intellectual disabilities, learning difficulties, and psychomotor delay, have been reported in 20% of subjects [3,18]. Dermatologic issues have been found in 90% of SDS patients [17]. Furthermore, other features including renal abnormalities, dental problems, endocrine dysfunction, hepatic complications, ocular manifestations, and solid tumors have been observed in a few cases [19].

The clinical variation among patients has encouraged several researchers to study the genotype–phenotype correlation. Very recently, a genotype–phenotype study was conducted by Thompson et al. on a large cohort of SDS patients (74 patients, 42 families). The study revealed a narrow genotypic spectrum that was not significantly associated with the phenotype [17]. This work confirmed the results of previous studies [20]. Other studies have succeeded in explaining some features of the hematological variability through somatic genetic events. Two particular types of karyotype instability have been reported, including i(7)(q10) (the isochromosome of the long arm of chromosome 7) and del(20)(q) (interstitial deletions of the long arm of chromosome 20). Interestingly, these two clonal anomalies, in the absence of additional clonal chromosomal abnormalities, are associated with a lower risk of developing MDS and/or AML [21,22,23].

Modern genetic techniques such as the Next Generation Sequencing (NGS) have led to a broader and more comprehensive understanding of the genetic basis of a large set of diseases, from rare Mendelian disorders to hereditary cancer [24,25]. Nowadays, clinical laboratories have adopted NGS as the gold standard for the diagnosis of hereditary disorders because of its analytic accuracy, high throughput, and potential for cost-effectiveness [26,27].

In Mendelian diseases, NGS-based technology is considered a very strong tool, not only for the detection of pathogenic mutations [28], but also to explain phenotypic variability [29,30]. NGS technology, in addition to bioinformatics based on the automatic implementation of the ACMG guidelines and artificially intelligent variant interpreters, now allows the analysis of thousands of variants simultaneously for a single patient using a single assay [31].

In this paper, we present two SDS siblings, both carrying the same *SBDS* pathogenic variants and showing relevant differences in their clinical presentation. We demonstrate that part of the clinical variability can be explained using WES and bioinformatics tools.

## 2. Materials and Methods

### 2.1. Case Presentation

#### 2.1.1. UPN42

This subject is a Caucasian female, and her weight at birth was 2870 g. In the first months of her life, she began to manifest a diarrheal alvus, and she was hospitalized several times for cough, fever, and dyspeptic symptoms, with features of pancreatic insufficiency. Since the first admission, a reduction in the number of neutrophils has been highlighted, and the hypothesis of autoimmune neutropenia was excluded. Gradually, over time, the symptoms of diarrhea with the characteristics of steatorrhea became more severe (5–7 discharges/day). At the age of 1 year, the hypothesis of cystic fibrosis was excluded via a sweat test, and SDS was diagnosed; oral replacement therapy with pancreatic extract was undertaken with good results. In regular follow-ups, up to the age of 21, the number of neutrophils was always lower than the average for her age. The hemoglobin values always remained within the normal range, and the count of reticulocytes was always appropriate for the hemoglobin levels. The platelet count was always lower than normal. Annual monitoring of bone marrow aspirates demonstrated poor or very poor cellularity, with notes of dyspoiesis in the myeloid precursors. The erythroid series in maturation was between 9 and 21% but it was not always observed; the share of mature lymphocytes reached a minimum of 44% and a maximum of 63%. Megakaryocytes were almost always absent. No blasts were found in any bone marrow examination and there was no evidence of any clonal evolution. She never requested blood transfusion or supportive therapy with G-CSF. At the age of 28, a FISH (Fluorescence In Situ Hybridization) examination confirmed the presence of a cell clone in 90% of the nuclei examined, containing a deletion of the centromeric region of chromosome 7 (7q11); this was consistent with the presence of i(7)(q10). At the age of 32, she developed Hodgkin lymphoma, and recovered after standard therapies; follow-ups are regular. The neuropsychological tests were normal, and the patient works as an employee.

#### 2.1.2. UPN43

This subject is a Caucasian male, and the brother of UPN42. His weight at birth was 3450 g. An obstetric ultrasonography test revealed a horseshoe kidney with bilateral pyelectasis, more evident on the left kidney. At the age of two months, features of seborrheic eczema with a significant atopic component were found, and at the age of 2.5 months, the patient developed chickenpox. At the same time, a growth delay was evident (4900 g, <3%; length 58 cm, <3%; head circumference 39.5 cm, <3%), and the appearance of greasy and foul-smelling stools was reported by his parents. Chemical-microscopic examination of the stool showed evidence of abundant neutral acids and fats and a tryptic power <2.5 UT; the sweat test was negative, and molecular confirmation of SDS was obtained. Weight recovery took place progressively, adapting the diet. At the age of 2 years, the hypothesis of pancreatic insufficiency was confirmed, and oral replacement therapy with pancreatic extract and fat-soluble vitamin supplements was undertaken. Weight increase improved, and infectious episodes (bronchitis and persistent cough) were sporadic, similar to the episodes of steatorrhea. At the age of 6 years, irregularities in the metaphyseal regions of the long bones, particularly at the level of the proximal and distal ends of the femurs and of the proximal ends of the tibias, were demonstrated. The metaphyseal regions appeared enlarged, frayed, and unevenly thickened. The femurs appeared curved with a medial concavity, and the coxo-femoral joints were regular. Slight metaphyseal irregularities were also appreciable in the humerus, radius and ulna. The ribs were short and squat. At the same age, he underwent orchidopexy due to cryptorchidism.

The hematological examinations consistently showed leukocyte counts low for his age. The hemoglobin values always remained in the normal range, while platelet counts remained low. Regular bone marrow monitoring, starting at the age of 15, always demonstrated poor cellularity with notes of dysmyelopoiesis. The erythroid series varied between 9% and 25%. The proportion of mature lymphocytes was always between 38 and 53%. Megakaryocytes have always been rare, and they were absent in the last check. No blasts have been found in any bone marrow examination, and no clonal evolution has been recorded. Similarly to his sister, he never requested blood transfusion or supportive therapy with G-CSF. FISH examination confirmed the presence of a cell clone in 90% of the nuclei examined, containing a deletion of the centromeric region of chromosome 7 (7q11), which is consistent with the presence of i(7)(q10). At last, minor facial dysmorphic features such as hypertelorism and wide nasal bridge were recorded. Records of family history show that he always needed additional support at school and that he can be defined as having a mild developmental delay, with problems in the areas of expressive language and memory; he has obtained a job within a national support program for people with minor handicaps.

### 2.2. Genetic Analysis

Genomic DNA was extracted from the peripheral blood of the two patients and their parents using the GenElute Blood Genomic kit (Sigma, St Louis, MO, USA) according to the manufacturer’s instructions. Whole-exome sequencing (WES) was performed as part of a project including 16 Italian SDS patients, using the HiSeq 1000 (Illumina, 2 × 100 bp). The patients, carrying biallelic pathogenic variants in the *SBDS* gene were identified by their Unique Patient Number (UPN), quoted in a previous study for our team [30]. Interesting variants found in the patients and their parents were validated using Sanger sequencing. Primers for variants of interest were designed using Primer3Plus (https://www.primer3plus.com/ 30 April 2022); PCR amplification was performed using a PrimeSTAR GXL DNA kit, and PCR products were purified using an enzymatic clean-up method (A’SAP-ArticZymes), followed by BigDye™ Terminator v3.1 Cycle Sequencing Kit. The samples were sequenced using a 3500 XL Series Genetic Analyzer.

### 2.3. Bioinformatics Analysis

#### 2.3.1. The Human Phenotype Ontology (HPO) Terms

Based on clinical information for both patients, the Human Phenotype Ontology (HPO) database was used to select the HPO terms describing our patients’ phenotypes (https://hpo.jax.org/app/ 22 April 2022) (Table 1).

#### 2.3.2. Filtering and Variant Prioritization

The Variant Call Format (VCF) files were generated via the ISAAC pipeline [32]; for data alignment and for variant calling, we used, respectively, the ISAAC aligner and ISAAC variant caller. We obtained a coverage mean of 65% and a coverage >20X corresponding to 91% of the complete exome sequences. For the annotation and the interpretation of the variants, we used enGenome eVai software (evai.engenome.com) which combines artificial intelligence with the American College of Medical Genetics (ACMG), AMP, and ClinGen guidelines to accurately report and classify genomic variants. Each genomic variation is evaluated in terms of effect, filtered based on phenotypes (HPO), and a score of pathogenicity for every single genomic variant is provided [31,33].

By using eVai, two different types of filters were applied:

Filter (1): Variants with good quality (QUAL ≥ 30) and high pathogenicity score (pathogenicity score ≥ 3) were selected for both patients; then, shared variants were excluded.

Filter (2): Variants with good quality (QUAL ≥ 30) and that were related to each HPO term described in Table 1 were selected. Variants with low or no impact on gene function (pathogenicity Score < 0), and deep intronic variants were excluded.

#### 2.3.3. Variant Interpretation

All variants resulting from filters 1 and 2 were screened according to their position, gnomAD frequency, mutation effect, mode of inheritance, mutation database data (ClinVar, LOVD, HGMD databases), literature, related diseases, and phenotypes using MalaCard, OMIM, and HPO. Additionally, in silico prediction tools including eVai pathogenicity score, ACMG, SIFT, MVP, FATHMM, Mutation Taster, M-CAP, and CADD were evaluated. For variants of interest, Uniprot (www.uniprot.org 10 January 2022) alignment tool was used to evaluate the amino acid conservation among different species such as humans, mice, rats, chickens, bovines, horses, and sheep.

The VCF file was also analyzed using DIVAs, an Explainable Artificial Intelligence (XAI) method for digenic variant identification and classification [34] (enGenome Srl). Briefly, this method, starting with patients’ phenotypes (as HPO list) and variants (in VCF format), exploits features at the variant level, gene–gene interaction level, and phenotype level to classify the digenic combination’s pathogenicity. Furthermore, XAI is used to investigate its digenic mechanism and subclassify each predicted pathogenic digenic combination as a True Digenic/Composite condition (whereby an interaction between the mutated genes triggers or exacerbates the phenotype), or a Dual Molecular Diagnosis (whereby two independent genetic events occur in the same individual, causing blended phenotypes).

## 3. Results

For both patients, the clinical diagnosis of SDS had been previously confirmed by the demonstration of two pathogenic variants in the *SBDS* genes c. [258 + 533_459 + 403del] and c. [258 + 2T > C], inherited from the father and the mother, respectively.

Using eVai, and according to the filter used, we selected:

Filter (1): 28 and 29 variants in UPN42 and UPN43, respectively.

(Supplementary Tables S1 and S2).

Filter (2): 14 variants in UPN42 and 82 variants in UPN43.

(Supplementary Tables S3 and S4).

All the variants were evaluated singularly, as mentioned in the Materials and Methods Section 2.3.3 (Variant Interpretation). eVai, of course, identified *SBDS* variants as relevant for patients’ phenotypes.

For UPN42, the single variant analysis did not disclose any variation, which could be related to the patient phenotype. Conversely, a *KMT2A* variant (c.10663G > A, p.Gly3555Ser) was found to be related to some of the HPO terms that describe UPN43, including developmental delay, horseshoe kidney, bone abnormalities, cryptorchidism, expressive-language delay, and minor facial dysmorphisms of hypertelorism and a wide nasal bridge (Table 1). Pathogenic variants in the *KMT2A* gene cause Wiedemann–Steiner Syndrome (WDSTS, OMIM #605130), an autosomal dominant disorder. Interestingly, the phenotype of this syndrome includes all HPO terms selected for UPN43, which are absent in UPN42.

**Figure 1:**
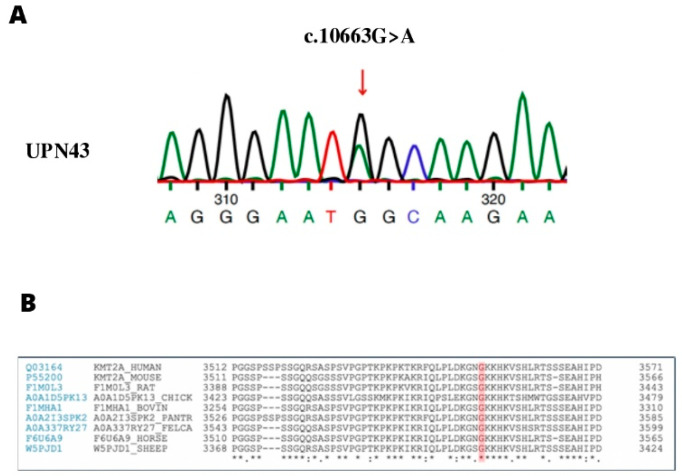
(**A**) Sanger sequencing confirmation. Part of the electropherogram illustrates the variant (c.10663G > A) in *KMT2A* in UPN43. (**B**) Uniprot alignment analysis shows that amino acid G (glycine) in position 3555 is highly conserved among different species such as humans, mice, rats, chickens, bovines, horses, and sheep.

The variant was validated in the trio using Sanger sequencing and demonstrated to be de novo with a heterozygous status (Figure 1A).

The de novo variant c.10663G > A in *KMT2A* is a novel one; it is unpublished, and no allele frequency is reported. The variant is classified as likely pathogenic according to the ACMG guidelines, and classified as damaging according to SIFT, MVP, FATHMM, Mutation Taster, M-CAP, and CADD.

Uniprot alignment analysis showed that amino acid G (glycine) in position 3555 is highly conserved among different species such as humans, mice, rats, chickens, bovines, horses, and sheep (Figure 1B).

As a further confirmation, the digenic combination involving *KMT2A* and *SBDS* genes in the UPN43 sample was predicted as pathogenic by DIVAs and subclassified as a likely Dual Molecular Diagnosis.

## 4. Discussion

Over the last decade, the introduction of WES and the implementation of Human Phenotype Ontology (HPO) has revolutionized the interaction between clinical diagnosis and molecular testing in monogenic and rare diseases. This combination allowed the time- and cost-effective sequencing of the whole exome to identify the genetic causes of a large number of syndromes with high clinical variability and genetic heterogeneity [35]. In medical genetic practice, WES has improved diagnostic yield by more than 25%, resulting in greater knowledge of the molecular mechanisms involved in Mendelian disorders [36].

SDS is a rare autosomal-recessive syndrome associated with three hallmark features including pancreatic insufficiency, neutropenia, and skeletal dysplasia. *SBDS* pathogenic variants are responsible for about 90% of reported cases [4]. However, the phenotypic spectrum of reported cases is wide, and the clinical variability among different patients, affected siblings, and in the same patient over time has been noted to be significant [17].

WES studies on SDS patients have been performed previously, either to identify new causative genes [8,10] or to explain the phenotypic variations found in some patients, including hematological problems [30,37,38].

Herein, we present two SDS siblings, (UPN42, female, and UPN43, male), who shared classical SDS features such as pancreatic insufficiency, hematological abnormalities (neutropenia, thrombocytopenia), and bone marrow hypocellularity. UPN43 showed additional characteristics not found in his sister, including skeletal problems (metaphyseal irregularities, metaphyseal-region enlargement, curved femurs, short ribs, and varus cervico-diaphyseal), renal abnormalities (horseshoe kidney with bilateral pyelectasis), recurrent infections, cryptorchidism, developmental delay, and minor dysmorphisms.

As an attempt to understand the clinical variability found in the two siblings, we analyzed WES data using the eVai and DIVAs platforms.

In UPN42, Hodgkin lymphoma (HL) was diagnosed; this disease has never been reported in any SDS patients, and the use of the eVai platform and DIVAs failed to identify any relevant variation, which is in keeping with the evidence of minor relevance of genetic factors in the development of HL. Recently, Weniger and a co-worker listed a number of single-nucleotide variants found to be associated with HL in a limited number of cases. However, none of the variants listed were found in UPN42 [39].

In UPN43, in addition to *SBDS* mutations, we identified and validated a heterozygous de novo missense variant located in exon 27 of the *KMT2A* gene (Figure 1A). The variant is classified as likely pathogenic according to the ACMG guidelines, and its pathogenicity score of eVai is 5, which is considered a high score. As the variant (c.10663G > A, p. Gly3555Ser) in *KMT2A* was not reported before, no information related to its frequency is available; two pathogenic variants have been identified close to the site where our variant was found, within 100 bp [40].

*KMT2A* is located on chr11q23.3, consists of 37 exons, and encodes a DNA-binding protein that methylates a lysine residue on histone H3 (H3K4) [41]. According to mouse studies, *KMT2A* is abundantly expressed in adult hippocampus neurons and is required for synaptic plasticity, cognition, complex behaviors, and long-term memory [42,43]. Pathogenic variants in *KMT2A* cause chromatin-remodeling deficiencies, which lead to widespread alterations in gene expression throughout development, resulting in abnormalities in multiple body systems [44]. The reported variants are located quite uniformly through the sequence of the gene, and most variants are clustered in exon 27, which is compatible with the fact that it is the longest exon [40,45].

Germline mutations in *KMT2A* cause autosomal-dominant Wiedemann–Steiner Syndrome (WDSTS, OMIM #605130), and, in the vast majority of cases, de novo mutations have been confirmed [46]. Since the initial association between WDSTS and *KMT2A* [47], more than 250 sequence variations have been described [45,47].

WDSTS is an exceptionally rare, <1/1,000,000, chromatinopathy disorder characterized mainly by distinctive facial dysmorphism, hypertrichosis cubiti, developmental delay, skeletal anomalies, short stature, psychomotor delay, horseshoe kidney, and ocular, cardiac, and dental manifestations [40,48,49].

The phenotypic spectrum of the disease is very wide; extensive clinical variability has been reported [46,47], and is further expanded through the finding of more cases via WGS and WES [50]. The WDSTS genotype–phenotype correlation is currently not fully understood, and the mild/unusual WDSTS presentations may be challenging to recognize [51].

Intellectual disability/psychomotor delay, usually mild-to-moderate, has been reported in 65–100% of cases. The prevalence of autism has been estimated to be 11.8%, but subjects without autism may also reveal behavioral abnormalities such as ADHD, anxiety, and emotional dysregulation [52].

Facial dysmorphisms are common (50–70%), and differ from one patient to another; they include hypertelorism, long and downslanting palpebral fissures, long eyelashes, a wide nasal bridge, low-set ears, a thin vermilion, micrognathia, and anomalies of the dentition [53,54]. Skeletal abnormalities such as hip dysplasia, delayed bone maturation, a short palm, fifth-finger clinodactyly, and small and buffy hands have been found in about half of patients. Hypertrichosis cubiti has been reported in approximately 60% of cases [39,54].

The wide clinical spectrum also includes a significantly higher risk of developing recurrent infections [55], congenital heart disorders (in 30% of cases, including septal defect and patent ductus arteriosus), ocular manifestations (such as ptosis, squint, lacrimal duct anomalies, and refractive errors) in about 50% of cases. [56]. Renal anomalies, including horseshoe kidney, pyelectasis, small or hypoplastic kidneys, and cryptorchidism, have been reported in about 30% of cases [40,45,49,57].

If we compare the phenotype of UPN43 with the classical SDS description and WDSTS, we note that hematological problems and pancreatic insufficiency are SDS-related and not reported in WDSTS.

Developmental delay, of variable severity, is found in about 65–76% of SDS cases [58], so it is part of the typical description of the syndrome. It is also reported in 65–100% of WDSTS patients [46,59]. At present, as the percentages of patients with developmental delay are roughly similar in both syndromes, so we cannot assign or define the specific contribution of each gene to this problem.

Although skeletal abnormalities are reported in about 50% of patients with WDSTS, the skeletal features of metaphyseal irregularities, curved femurs, and short ribs, present in UPN43, are considered classical for patients with *SBDS* mutations and more compatible with SDS than WDSTS. In the latter syndrome, the typical skeletal abnormalities are hip dysplasia, delayed bone maturation, and short palm and fifth-finger clinodactyly [40,59].

Recurrent infection episodes, present in UPN43, are typical for SDS as a consequence of neutropenia, which is mild-to-moderate, on different occasions. Infections have been reported in both syndromes, and we have no evidence, at present, of any interaction between *SBDS* and *KMT2A*, which could be related to the worsening of infections.

Conversely, horseshoe kidney with/without pyelectasis was never reported in any SDS patient (PubMed and Google Scholar search, June 2022, using horseshoe kidney AND Shwachman-Diamond Syndrome), and is found in approximately 0.25% of the general population; moreover, it is more commonly reported in WDSTS, with six patients showing this malformation [40,49,59,60,61].

Similarly, cryptorchidism, not reported in patients with *SBDS* mutations (PubMed and Google Scholar search, June 2022, using cryptorchidism AND Shwachman-Diamond Syndrome), is present in UPN 43, its prevalence in the normal population is 1.7%, and observed in about 20–35% of WDSTS cases [49,61,62]. However, despite the fact that cryptorchidism has been reported in some WDSTS patients, it has never been associated with *KMT2A* mutations; instead, it has been associated with *TASP1,* which is a regulator of *KMT2A* [63].

The features of facial dysmorphisms, horseshoe kidneys, pyelectasis, and cryptorchidism are not hallmarks of SDS; instead, they are more likely related to WDSTS. Therefore, we postulate that the *KMT2A* variant, found in UPN43, for which there is in silico evidence of it being pathogenic, has a concomitant and co-occurring clinical effect. This dual molecular effect, supported by in silico prediction via the DIVAs tool, could help to understand some of the clinical variations found among the siblings.

*KMT2A* was previously known as MLL (Mixed-Lineage Leukemia) because of its recognition as a frequent target of somatic rearrangements in acute leukemia [64]. Until now, no patients with WDSTS were reported to develop any type of hematological malignancy, so there is no evidence that the mutation we found acts as a risk factor for UPN43.

## 5. Conclusions

The reporting of these cases underlines the need for extended genome analysis in patients affected with SDS showing unexpected or unusual phenotypes. The availability of deep genome analysis and artificial-intelligence-based bioinformatics tools suggest that the well-known clinical variability reported in Mendelian diseases could be partially explained by these techniques. These new data are likely, in the future, to also be relevant for personalized medicine and therapy in selected cases or groups of patients.

## Figures and Tables

**Table 1:** HPO ^1^ terms that describe UPN42 and UPN43 phenotypes.

UPN42	UPN43
HP:0001875 Neutropenia	HP:0000085 Horseshoe kidney
HP:0005528 Bone marrow hypocellularity	HP:0001875 Neutropenia
HP:0001738 Exocrine pancreatic insufficiency	HP:0005528 Bone marrow hypocellularity
HP:0001873 Thrombocytopenia	HP:0001738 Exocrine pancreatic insufficiency
HP:0040088 Abnormal lymphocyte count	HP:0003025 Metaphyseal irregularity
HP:0012189 Hodgkin lymphoma	HP:0012758 Neurodevelopmental delay
	HP:0001873 Thrombocytopenia
	HP:0000028 Cryptorchidism
	HP:0002719 Recurrent infection
	HP:0000431 Wide nasal bridge
	HP:0000316 Hypertelorism
	HP:0002474 Expressive-language delay

^1^ HPO: Human Phenotype Ontology.

## Data Availability

The data that support the findings of this study are available from the corresponding author upon reasonable request.

## Supplementary Materials

The following supporting information can be downloaded at https://www.mdpi.com/article/10.3390/genes13081314/s1, Table S1: variants with good quality (QUAL ≥ 30) and high pathogenicity score (pathogenicity score ≥ 3) found in UPN42; Table S2: variants with good quality (QUAL ≥ 30) and high pathogenicity score (pathogenicity score ≥ 3) found in UPN43; Table S3: variants with good quality (QUAL ≥ 30) and that are related to each HPO term described in Table 1 for UPN42; Table S4: variants with a good quality (QUAL ≥ 30) and that are related to each HPO term described in Table 1 for UPN43.
